# Supplementary material for: Assessing the prognostic scores for the prediction of the mortality of patients with acute-on-chronic liver failure: a retrospective study
Source: PeerJ. 2020 Sep 15;8:e9857. doi: 10.7717/peerj.9857 (PMC7500347; doi:10.7717/peerj.9857)
Supplement: Supplemental Information 2 [file peerj-08-9857-s002.docx]

| Cause of death | 28-days (n=47) | 3-months (n=58) | 6-months (n=61) |
| --- | --- | --- | --- |
| Respiratory failure, n (%) | 12(25.5%) | 12(20.7%) | 12(19.7%) |
| Hemorrhagic shock, n (%) | 15(31.9%) | 18(31%) | 18(29.5%) |
| Infectious shock, n (%) | 4(8.5%) | 6(10.3%) | 6(9.8%) |
| Hepatic encephalopathy, n (%) | 8(17%) | 13(22.4%) | 15(24.6%) |
| cardiogenic shock, n (%) | 3(6.4%) | 3(5.2%) | 3(4.9%) |
| Liver failure, n (%) | 4(8.5%) | 4(6.9%) | 4(6.6%) |
| Uncertain, n (%) | 1(2.1%) | 2(3.4%) | 3(4.9%) |

Supplement table 1: Cause of death of patients with ACLF
